# Phenolic Constituents Drive Antimicrobial and Antibiotic-Enhancing Activities of *Cannabis sativa* Seed Extracts Obtained by Two Extraction Methods

**DOI:** 10.3390/plants15010027

**Published:** 2025-12-21

**Authors:** Doris Floares (Oarga), Diana Obistioiu, Anca Hulea, Ersilia Alexa, Marinel Nicolae Horablaga, Adina Berbecea, Florin Crista, Cristina Dehelean, Isidora Radulov

**Affiliations:** 1 Faculty of Agriculture, University of Life Sciences “King Michael I” from Timisoara, Calea Aradului 119, 300645 Timisoara, Romania; doris.oarga@usvt.ro (D.F.); anca.hulea@usvt.ro (A.H.); marinel_horablaga@usvt.ro (M.N.H.); adina_berbecea@usvt.ro (A.B.); florin_crista@usvt.ro (F.C.); isidora_radulov@usvt.ro (I.R.); 2 Faculty of Food Engineering, University of Life Sciences “King Michael I” from Timisoara, Calea Aradului 119, 300645 Timisoara, Romania; ersiliaalexa@usvt.ro; 3 Agricultural Research and Development Station, Lovrin, Principala Street No. 200, 307260 Lovrin, Romania; 4 Faculty of Pharmacy, “Victor Babes” University of Medicine and Pharmacy, 2 Eftimie Murgu Square, 300041 Timisoara, Romania; cadehelean@umft.ro

**Keywords:** antibiotic synergy, *Cannabis sativa*, hemp seed extracts, HPLC phenolics, plant antimicrobials, ultrasound-assisted extraction (UAE)

## Abstract

Hemp seeds (*Cannabis sativa* L.) are a rich source of phenolic compounds with antioxidant and antimicrobial potential. Still, their genotype-dependent variability and ability to enhance antibiotic efficacy remain insufficiently explored. This study compared three Romanian hemp seed cultivars (Lovrin 110, Silvana, and LV 585) extracted by conventional hydroalcoholic extraction (CE) and ultrasound-assisted extraction (UAE) to evaluate their phenolic composition, antimicrobial effects, and synergistic interactions with amoxicillin and miconazole. HPLC identified genotype- and method-dependent differences, with UAE extracts showing substantially higher levels of epicatechin, quercetin, rosmarinic acid, resveratrol, and ferulic acid. These patterns showed stronger antimicrobial inhibition against Gram-positive and Gram-negative bacteria and yeasts, confirmed by MIC, fold-reduction, and percent enhancement assays. The most pronounced synergy occurred in *Streptococcus pyogenes*, *Staphylococcus aureus*, *Bacillus cereus*, and *Candida albicans*. PCA revealed two dominant phenolic-activity axes: a rosmarinic/resveratrol/ferulic axis associated with potent inhibition in *Escherichia coli* and *C. albicans*, and a quercetin-driven axis linked to Gram-positive bacteria. Overall, UAE extracts displayed superior phenolic enrichment and bioactivity, demonstrating that specific phenolic structures—not total phenolic content—govern antimicrobial performance and antibiotic-enhancing potential in hemp seed extracts.

## 1. Introduction

*Cannabis sativa* L. (hemp) is an herbaceous, anemophilous species in the *Cannabaceae* family. It is considered one of the oldest domesticated crops [1]. Due to its long history of use, it is now seen as a versatile, sustainable crop with a relatively low environmental impact and significance across sectors such as agriculture, phytoremediation, food and feed production, cosmetics, construction materials, and pharmaceuticals [2,3,4,5]. This species offers a wide array of valuable products, such as fibres and shives for technical and building applications, insulation materials, seeds, flours, and oils with notable nutritional and functional qualities, as well as bioactive molecules of pharmacological interest.

Beyond their fundamental nutritional value, namely, 25–35% lipids with a well-balanced, distinctive fatty-acid profile, 20–25% easily digestible protein rich in essential amino acids, and about 20–30% carbohydrates, hemp seeds are also rich in natural antioxidants and bioactive compounds such as peptides, phenolic compounds, tocopherols, carotenoids, and phytosterols [6,7].

Hemp seed has historically served as a medicinal resource, used to address a range of conditions such as arthritis, asthma, menstrual discomfort [8], atopic dermatitis [9] cancer [10], hypertension [11], and other inflammatory diseases [12].

Infectious diseases remain a major global cause of illness and death, with millions of fatalities each year linked to the rise in antibiotic-resistant bacterial strains [13]. Given this context, there is a growing interest in examining alternative strategies to prevent and manage microbial infections. Bioactive compounds from plants—such as alkaloids, tannins, and polyphenols—are being investigated both as antimicrobial agents and as modulators of antibiotic resistance [14,15,16].

One complementary approach is the combined use of medicinal plants with standard antibiotics to restore the effectiveness of drugs against resistant strains. Plant-based remedies have been used since antiquity and remain relevant for both therapeutic and preventive purposes; in particular, certain phytochemicals can act synergistically with antibiotics, boosting their antimicrobial potency and allowing antibiotics to act even against bacteria that had previously shown resistance [17]. The antimicrobial activity of plant polyphenols heavily depends on their chemical structure—including the number and position of hydroxyl, methoxy, and prenyl groups, the pattern of π-electron delocalisation, steric effects, and whether the molecule is glycosylated [18]. Small structural changes, like swapping –OH and –OCH_3_ positions, can influence whether a flavonoid is effective or ineffective against bacteria such as *Escherichia coli* and *Staphylococcus aureus* [19]. Typically, unglycosylated forms, such as quercetin, exhibit greater antimicrobial potency than their glycosylated counterparts, primarily due to fewer free –OH groups and enhanced lipophilicity, which promote more effective interactions with microbial membranes [20].

A wide range of traditional and emerging extraction techniques can be used to isolate bioactive compounds from plant material. The choice of method and solvent depends on the nature and location of the phenolic compounds in the sample. Ultrasonic-assisted extraction (UAE) is considered particularly suitable for recovering plant bioactives because it is quick, requires relatively little solvent, and is effective even for heat-sensitive molecules [21,22]. It is also considered an environmentally friendly method [23]. Compared to traditional extraction techniques, UAE prevents the degradation of thermolabile compounds, decreases solvent and energy consumption, shortens extraction duration, and can improve overall yield [24].

This work investigates three Romanian *Cannabis sativa* seed genotypes—Lovrin 110 (HSL), Silvana (HSS), and the dioecious line LV 585 (HSLV)—to determine how the extraction method influences their bioactive potential. Conventional hydroalcoholic extraction (CE) and ultrasound-assisted extraction (UAE) are compared in terms of their impact on phenolic composition, individual polyphenol profiles, and antimicrobial activity. In addition, the study examines synergistic interactions between hemp seed extracts and sub-inhibitory concentrations of antibiotics and antifungals. By integrating genotype-specific variability with extraction-dependent differences, this research provides a microbiology-centred perspective on Romanian hemp seeds as promising natural antimicrobial agents and potential enhancers of antibiotic efficacy.

## 2. Results

### 2.1. Individual Polyphenols by HPLC Analysis

Table 1 shows the individual polyphenols identified by HPLC across six samples.

Among phenolic acids, gallic acid was detected in the HSSUAE sample at 178.1 µg/g; caffeic acid in the HSLVCE (4.79 µg/g) and HSSUAE (3.60 µg/g) samples; and β-resorcylic acid in the HSSCE (10.27 µg/g) and HSLUAE (18.02 µg/g) samples. Coumaric acid, also a phenolic acid, was detected in all three samples obtained through conventional extraction, with levels between 3.50 µg/g and 4.32 µg/g, showing no significant (*p* > 0.05) difference between HSLCE and HSLVCE, but a higher value of 6.80 µg/g in the HSLUAE sample; significant differences (*p* < 0.05) exist between the HSLCE and HSLUAE samples. Ferulic acid, another phenolic acid, was present in all samples except HSSUAE, with concentrations ranging from 3.81 µg/g to 4.72 µg/g. Notably, among ultrasound-assisted extracts, significant differences (*p* < 0.05) were observed. Rosmarinic acid was detected in all samples, with concentrations ranging from 1.82 µg/g to 11.24 µg/g. It demonstrated significant differences (*p* < 0.05) among the samples obtained through identical extraction methods, as well as between the HSLCE and HSLUAE samples, which were produced using different extraction techniques. Non-significant differences (*p* > 0.05) between the two extraction methods were observed for the HSSCE vs. HSSUAE samples and for the HSLVCE vs. HSLVUAE samples. Regarding flavonoids, epicatechin was identified in only one of the conventional extraction samples (HSSCE, 18.45 µg/g), but in all ultrasound-assisted samples. The concentrations followed the order HSLVUAE > HSSUAE > HSLUAE, with notable differences (*p* < 0.05). The HSSCE and HSSUAE samples showed significant differences (*p* < 0.05), where ultrasound extraction yielded higher values than conventional methods. Quercetin was present in all samples, with levels ranging from 4.15 µg/g to 20.53 µg/g, and significant differences (*p* < 0.05) were observed between the conventional and ultrasound-assisted extracts. Resveratrol from the stilbene class ranged from 1.41 to 13.29 µg/g, showing significant differences (*p* < 0.05) between conventional and ultrasound extraction.

### 2.2. Antimicrobial Activity

#### 2.2.1. Bacterial/Mycelial Inhibitory Evaluation

**Figure 1:**
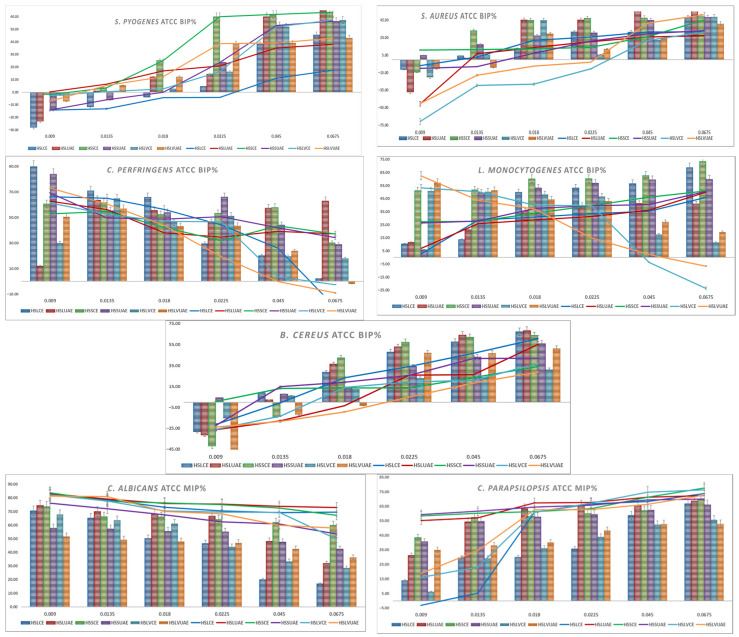
Graphical representation of BIP% of Gram-positive and fungal ATCC strains.

**Figure 2:**
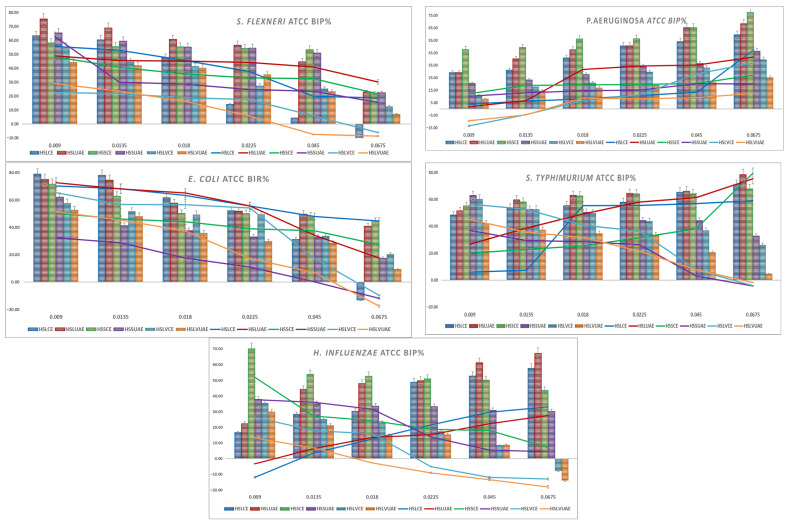
Graphical representation of BIP% of Gram-negative ATCC strains.

Graphical representations of the BIP% for Gram-positive and Gram-negative bacteria are presented in Figure 1 and Figure 2. Lines are the BIP% of single extracts, and cluster columns are representations of the BIP% of antibiotics potentiated by extracts.

*S. pyogenes* showed a clear dose-dependent response. At low concentrations (0.009–0.0135 mg/mL), most extracts caused negative or minimal inhibition, while HSLUAE and HSSCE showed the earliest positive effects. From 0.018 mg/mL upward, all extracts became inhibitory, with HSSCE, HSLUAE and HSLVUAE increasing most rapidly and reaching 50–70% inhibition at the highest doses. The addition of amoxicillin markedly enhanced activity across all genotypes. At 0.0135–0.0225 mg/mL, several extracts showed 15–30% greater inhibition than the extract alone, indicating strong synergy, especially for HSLUAE, HSSCE, and HSLVUAE. High-dose combinations maintained potent inhibition (55–68%) with no antagonism observed. The MIC values for *S. pyogenes* ranged from 0.009 to 0.045 mg/mL, with the highest sensitivity observed for HSLUAE, HSLUAE+A, HSSCE+A, HSSUAE+A, HSLVCE+A, HSLVUAE, and HSLVUAE+A (all 0.009 mg/mL), whereas HSLCE showed the highest MIC (0.045 mg/mL). Overall, *S. pyogenes* was highly responsive to phenolic composition, and UAE extracts—particularly HSLUAE—showed the most substantial and antibiotic-enhancing effects.

Regarding *S. aureus*, the ATCC strain showed dose-dependent inhibition. At low concentrations (0.009–0.0135 mg/mL), most extracts produced negative BIP values, while HSSCE was the only sample showing early inhibition. Starting at 0.018 mg/mL, all extracts became inhibitory, reaching 25–45% at high doses, with HSLVUAE and HSSCE being the most active; the addition of amoxicillin strongly enhanced activity. At 0.0135–0.0225 mg/mL, several extracts gained 20–30% inhibition compared to the extract alone, indicating strong synergy; high-dose combinations maintained 45–69% inhibition with no antagonism. Synergy patterns corresponded to phenolic composition: quercetin-rich HSLVUAE and caffeic/gallate-rich HSSCE produced the highest antibacterial and synergistic effects. For *S. aureus*, MIC values ranged between 0.009 and 0.045 mg/mL. Most extracts and combinations showed MICs of 0.009–0.0135 mg/mL, while only HSLCE reached 0.0135 mg/mL, and HSLVCE/HSLVUAE+A displayed the highest MIC (0.045 mg/mL).

*C. perfringens* was strongly inhibited by all hemp extracts at low concentrations, reaching 60–90% BIP for HSLCE, HSSUAE, and HSLVUAE at 0.009 mg/mL. Inhibition gradually decreased with increasing extract concentration, with HSLCE showing the sharpest decline, while HSSCE/HSSUAE maintained moderate activity (40–55%) across the mid-range. HSLVCE and HSLVUAE showed variable, often low or negative inhibition at the highest concentrations. The addition of an antibiotic improved activity only modestly (5–15%) and predominantly at low doses, particularly for HSLCE, HSLUAE, and HSSCE. Synergy was weak, with most combinations behaving additively and no clear antagonism detected. Overall, *C. perfringens* responded best to Lovrin CE and Silvana UAE extracts at low concentrations, while high-dose effects diminished across genotypes. *C. perfringens* showed identical MIC values across all extracts and combinations, with a uniform MIC of 0.009 mg/mL, indicating high and consistent susceptibility.

*L. monocytogenes* showed a clear dose-dependent inhibitory response across all hemp extracts. At the lowest concentration (0.009 mg/mL), activity remained weak to moderate (5–13%), with HSLVCE and HSLVUAE reaching the highest BIP% values (≈50–62%). From 0.018 mg/mL onward, all extracts exhibited progressively more potent inhibition, with HSLCE, HSLUAE, HSSCE, and HSSUAE increasing steadily from ≈35–60% at 0.018–0.045 mg/mL. At the highest concentration (0.0675 mg/mL), HSLCE (68.7%), HSSCE (73.5%), and HSSUAE (59.8%) showed the most potent inhibition, confirming robust dose-responsiveness. With amoxicillin, inhibition improved across all extracts. At 0.009–0.0135 mg/mL, BIP% values increased substantially (≈2–28%), and synergy was most evident in HSLUAE (25–28%), HSSCE (27–33%), and HSLVCE (53–50%). Higher concentrations (0.018–0.0675 mg/mL) maintained elevated inhibition, reaching 46–51% at 0.0675 mg/mL for HSLCE, HSLUAE, HSSCE and HSSUAE. No antagonism was observed at any dose. *L. monocytogenes* showed uniform sensitivity across all extracts and combinations, with a consistent MIC of 0.009 mg/mL for every sample tested. *L. monocytogenes* responded strongly to increasing extract concentration, with CE and UAE extracts of all cultivars—particularly HSSCE and HSLCE—producing the highest inhibition. At the same time, antibiotic addition provided consistent additive enhancement.

*B. cereus* exhibited weak or negative inhibition at the lowest concentrations for most extracts (–45% to –5%), particularly for HSLCE, HSLUAE, and HSSCE. Activity increased steadily with dose, reaching 50–70% BIP at the highest concentrations. The most substantial effects were observed for HSLCE, HSLUAE, and HSSUAE, all of which exceeded 60% BIP at 0.0675 mg/mL, whereas HSLVCE and HSLVUAE showed moderate inhibition (30–50%). The antibiotic–extract mixtures produced modest improvement relative to extracts alone, especially for HSLCE, HSLUAE, and HSSUAE at mid–high concentrations (15–25% gain). Synergy remained weak, with enhancement values compatible with additive effects and no antagonism. The MIC values for *B. cereus* ranged from 0.009 to 0.0225 mg/mL, with the lowest MIC (0.009 mg/mL) recorded for HSSCE, HSSCE+A, and HSSUAE+A, whereas HSLUAE and HSLVUAE+A showed the highest MIC (0.0225 mg/mL). *B. cereus* responded best to Lovrin CE/UAE and Silvana UAE, showing a clear dose-dependent inhibition pattern.

*S. flexneri* showed high baseline inhibition across all extracts, with strong activity even at 0.009 mg/mL (55–75%). Inhibition gradually decreased with increasing concentration, stabilising at 40–55% for most extracts and dropping below 10% only for HSLVCE and HSLVUAE at the highest dose. HSLUAE, HSSCE, and HSSUAE maintained the strongest activity throughout the concentration range, while HSLVCE consistently exhibited the weakest inhibition. In the presence of the antibiotic, inhibition remained consistently high (approximately 45–70% at low doses), with minimal differences compared with the extracts alone, indicating an additive rather than synergistic interaction. No antagonism was observed. Overall, *S. flexneri* responded uniformly to all phenolic profiles, with no significant enhancement resulting from the addition of the antibiotic. For *S. flexneri*, all extracts and antibiotic combinations displayed a consistent MIC of 0.009 mg/mL.

*P. aeruginosa* showed moderate inhibition across all extracts, increasing steadily with concentration. HSSCE and HSLUAE were consistently the most active, reaching 60–75% inhibition at the highest doses, while HSLVUAE remained the weakest. Low-dose values ranged from 10–30%, increasing to 40–70% at 0.045–0.0675 mg/mL. Antibiotic combinations caused minor (5–10%) increases or none, indicating additive effects, not synergy. No antagonism was observed. *P. aeruginosa* showed a concentration-dependent response with limited enhancement due to its membrane resistance. MIC values ranged from 0.009 to 0.018 mg/mL, mostly at 0.009 mg/mL, with HSLVCE+A and HSLVUAE+A higher at 0.018 mg/mL.

*E. coli* showed potent baseline inhibition for all extracts at low concentrations (70–78%), with HSLCE, HSLUAE, and HSSCE being the most active. Inhibition gradually declined with increasing concentration, stabilising at 40–55% by 0.0225 mg/mL and then dropping further at the highest doses, particularly for HSLVCE and HSLVUAE, which fell below 0. HSLCE and HSLUAE maintained the highest activity across the full range. Antibiotic combinations produced only modest improvements (typically 5–10%) and in some extracts no enhancement at all, indicating additive rather than synergistic interactions. No antagonism was detected. Overall, *E. coli* responded uniformly across genotypes, with UAE and CE extracts of HSL showing the strongest and most consistent activity. The MIC values for *E. coli* ranged from 0.009 to 0.018 mg/mL. Most extracts showed MICs of 0.009 mg/mL, while HSLCE+A and HSLVCE+A showed the highest MIC (0.018 mg/mL).

*S. typhimurium* showed consistently high inhibition across all extracts, with 48–63% activity at the lowest concentration and values reaching 65–78% at the highest doses. HSLUAE, HSSCE, and HSLVCE were the strongest across all concentrations, while HSLVUAE showed the weakest inhibition, dropping to 0.0675 mg/mL. The addition of an antibiotic produced only small increases in inhibition, typically 5–10%, and, in several cases, no enhancement or even a slight decrease, indicating an additive, not synergistic, interaction. No antagonism was observed. Overall, *S. typhimurium* responded uniformly to the extracts, with minimal benefit from antibiotic supplementation. All extracts and antibiotic combinations displayed a uniform MIC of 0.009 mg/mL for *S. typhimurium*, indicating high sensitivity.

*H. influenzae* showed potent inhibition at low and intermediate concentrations, with HSSCE, HSLUAE, and HSSUAE producing 35–70% inhibition at 0.009–0.0225 mg/mL. Activity levels exhibited a slight increase at higher dosages for the majority of extracts, with the exception of HSLVCE and HSLVUAE, which demonstrated a reduction to negative values at 0.0675 mg/mL. Overall, HSLCE, HSLUAE, and HSSCE maintained the most consistently active profiles. The inclusion of antibiotics resulted in only modest improvements (generally less than 10%), and in certain extracts, no enhancement was observed, suggesting additive interactions rather than synergistic effects. No antagonism was detected. These results show that *H. influenzae* responds well to hemp seed phenolics, but gains minimal benefit from antibiotic supplementation. *H. influenzae* demonstrated consistent sensitivity to all extracts, with identical MIC values of 0.009 mg/mL across all treatments.

*C. parapsilosis* showed potent inhibition across all extracts, starting at 40–60% at the lowest concentration and reaching 65–75% at the highest doses. UAE extracts (HSLUAE, HSSUAE, HSLVUAE) and HSSCE showed the highest and most consistent activity across all concentrations. At the same time, HSLCE remained the weakest at low doses but improved substantially at higher levels. In combination with miconazole, inhibition increased modestly (5–10% on average), indicating additive effects with weak synergy. No antagonism was observed. Overall, *C. parapsilosis* was strongly inhibited by all extracts, with only minor enhancements from antifungal supplementation. *C. parapsilosis* displayed identical MICs of 0.009 mg/mL for all extracts and combinations, indicating uniformly high sensitivity.

*C. albicans* showed an evident dose-dependent decline in inhibition across all hemp extracts. At the lowest concentration (0.009 mg/mL), all extracts demonstrated high activity (≈70–84%), with HSLUAE and HSLVCE showing the strongest inhibition (≈74–83%). As the concentration increased, inhibition progressively diminished across all extracts. At 0.018 mg/mL, the values decreased to a range of 50–68%, and by 0.0225 mg/mL, most extracts exhibited inhibition levels between 43–66%. The minimal activity was observed at 0.0675 mg/mL, where inhibition declined to between 16–53%, with HSLCE and HSLUAE demonstrating the most pronounced reductions (approximately 17–32%). Conversely, the antibiotic combination (extract plus miconazole) consistently maintained high levels of inhibition throughout the entire concentration spectrum. At 0.009 mg/mL, all extracts achieved 76–83% MIP, and at 0.0135–0.018 mg/mL, values remained elevated at approximately 72–79%. Even at the highest concentration (0.0675 mg/mL), inhibition persisted robustly across most extracts, ranging from approximately 49–73%, with HSLUAE and HSLCE showing the most notable improvements relative to the extracts alone. This demonstrates an apparent additive-to-moderate synergistic effect, particularly noticeable as extract-alone activity declines at higher doses. For *C. albicans*, all extracts and combinations produced a uniform MIC of 0.009 mg/mL, showing strong susceptibility.

Overall, *C. albicans* responded strongly at low extract concentrations but showed a dose-dependent reduction in inhibition when extracts were used alone. The presence of miconazole stabilised and enhanced antifungal performance, maintaining high MIP% values across all doses, with the UAE extracts (especially HSLUAE) producing the most consistent effects.

Across all strains, the heatmap reveals two main trends: (i) UAE extracts (HSLUAE, HSSUAE, HSLVUAE) cluster towards high inhibition (red), indicating richer phenolic content. (ii) Conventional extracts (HSLCE, HSSCE, HSLVCE) show variable inhibition, with some strains high and others moderate/low inhibition.

**Figure 3:**
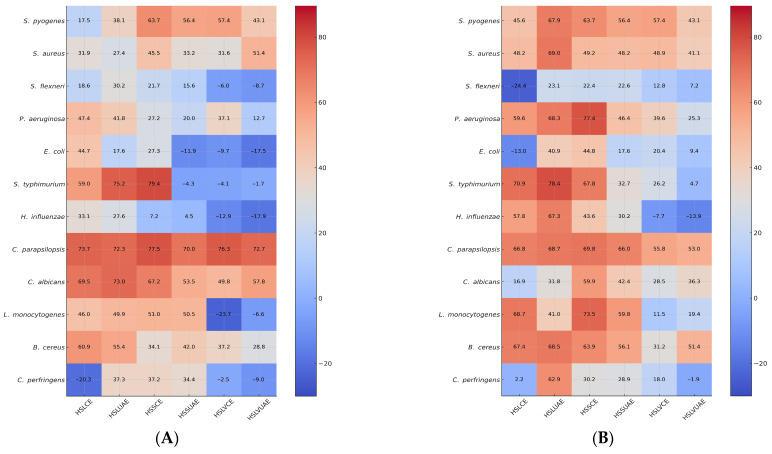
Heatmap representation of BIP% and MIP% at a final test concentration of 0.0675 mg/mL of single extracts or mixture with amoxicillin (for bacteria) or miconazole (for yeasts). Values represent the Bacterial/Fungal Inhibition Percentage (BIP%/MIP%) produced by the hemp seed extracts (**A**) and mixtures of hemp seed extracts with amoxicillin (for bacteria) or miconazole (for yeasts) (**B**)—HSLCE, HSLUAE, HSSCE, HSSUAE, HSLVCE, and HSLVUAE—at a final test concentration of 0.0675 mg/mL. Rows correspond to inhibition against each microbial strain (*S. pyogenes*, *S. aureus*, *L. monocytogenes*, *B. cereus*, *C. perfringens*, *S. flexneri*, *P. aeruginosa*, *E. coli*, *S. typhimurium*, *H. influenzae*, *C. parapsilosis*, *C. albicans*). Colour intensity reflects inhibitory strength (blue = low/negative inhibition; red = strong inhibition), allowing comparison with extract-only activity and visualisation of additive/synergistic/antagonistic trends.

At the final test concentration of 0.0675 mg/mL, the heatmap (Figure 3) reveals clear extract- and strain-dependent antimicrobial patterns. UAE extracts (HSLUAE, HSSUAE, HSLVUAE) consistently cluster in the high-inhibition (red) zone, reflecting their richer phenolic profiles, while CE extracts display more variable intensities. Gram-positive strains such as *S. pyogenes*, *S. aureus*, *L. monocytogenes*, and *B. cereus* show strong to moderate inhibition across all extracts, with UAE samples generally producing the most intense signals. Gram-negative bacteria show diverse responses: *S. flexneri*, *S. typhimurium*, and *E. coli* have high inhibition; *P. aeruginosa* and *H. influenzae* are more moderate, reflecting membrane resistance. *C. parapsilosis* shows consistent strong inhibition, while *C. albicans* has reduced activity at this concentration, leading to lighter heatmap regions.

The extract + antibiotic/antifungal combinations generally shift heatmap colours toward darker red, indicating additive or synergistic enhancement. The strongest intensifications are observed for *S. pyogenes* + amoxicillin and *C. albicans* + miconazole, where combinations restore or exceed the inhibition observed with the extract alone, even when single-extract activity declines at higher concentrations. For most Gram-negative species, particularly *P. aeruginosa* and *H. influenzae*, antibiotic addition produces only modest colour shifts, confirming predominantly additive interactions. Overall, the heatmap indicates that antimicrobial efficacy and enhancement trends are predominantly influenced by the extraction technique and phenolic content, with UAE extracts exhibiting the most consistent and potent inhibition profiles across the microbial spectrum.

#### 2.2.2. Fold Reduction in MIC

The fold-reduction analysis (Table 2) shows that synergy occurred selectively and was strongly dependent on genotype and extraction method. Moderate synergy (2–4-fold reduction) was observed mainly for *S. pyogenes*, *S. aureus*, and *B. cereus*, with the most notable effects produced by HSSUAE+A (3.8-fold for *S. pyogenes*; 2.0-fold for *S. aureus*) and HSLVCE+A (3.3-fold for *S. pyogenes* and *S. aureus*). In contrast, *C. perfringens*, *S. flexneri*, *E. coli*, *S. typhimurium*, *H. influenzae*, *C. albicans*, and *C. parapsilosis* consistently showed a fold-reduction value of 1.0, indicating additive interactions without true synergy. No antagonism (values < 1) was observed for any strain.

Overall, the fold-reduction data confirm that synergistic interactions appear primarily in selected Gram-positive bacteria, particularly when extracts rich in flavonoids or phenolic acids are combined with amoxicillin. For most Gram-negative bacteria and fungi, however, the combinations behaved additively, reflecting their higher intrinsic resistance and reduced susceptibility to synergy.

#### 2.2.3. Antibiotic/Antifungal % Enhancement

The % enhancement analysis in Table 3 shows that all extract–antibiotic/antifungal combinations produced additive or synergistic improvements, with no antagonistic effects. The strongest enhancement values were recorded for *C. perfringens*, *S. flexneri*, *E. coli*, *S. typhimurium*, *H. influenzae*, and *C. albicans*, where several extracts increased inhibition by 50–80%, indicating that even strains with high baseline susceptibility benefited from extract-mediated potentiation. *C. albicans* showed high enhancement across all extracts, confirming a strong additive-to-synergistic interaction with miconazole that countered the natural decline in extract-alone activity at higher concentrations.

For Gram-positive bacteria, moderate-to-strong enhancement (up to 50–57%) was seen for *L. monocytogenes* and *B. cereus*, especially with HSSCE+A, HSLVUAE+A, and HSLVCE+A, indicating specific phenolic profiles boost amoxicillin activity. In contrast, *S. pyogenes* and *S. aureus* showed lower enhancement values (generally < 35%), consistent with the strong intrinsic activity of the extracts themselves, which leaves less room for improvement by the antibiotic. Overall, the enhancement patterns indicate that phenolic-rich extracts mainly boost antimicrobial effectiveness via additive effects, with some instances of moderate synergy, especially against *C. albicans*, *C. perfringens*, and certain Gram-negative strains.

### 2.3. Multivariate Correlation Patterns of the Compounds Based on PCA

**Figure 4:**
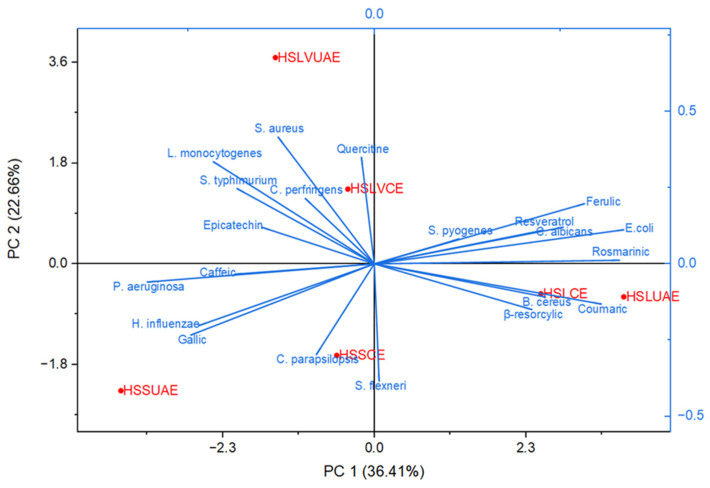
Principal Component Analysis (PCA) illustrating the correlation structure between the individual polyphenols identified in the hemp seed extracts and the inhibition profiles of the tested microbial strains.

**Figure 5:**
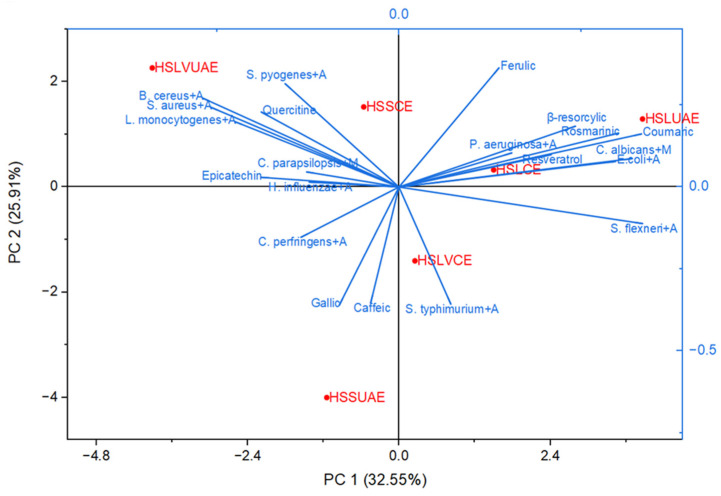
Principal Component Analysis (PCA) showing the relationships between individual polyphenols identified in the hemp seed extracts and the microbial strains treated with amoxicillin/miconazole agents.

A multi-parametric statistical approach based on PCA (Figure 4 and Figure 5) was employed to further examine the relationships among individual polyphenols, microbial strains, and microbial strains treated with antimicrobial/antifungal agents. Using a linear correlation matrix, PCA was performed on the mean values of the measured variables to identify the parameters that contributed most significantly to the overall variation in the dataset.

The first two components—PC1 (36.41%) and PC2 (22.66%)—explained 59.07% of the total variance, capturing the dominant compositional and functional patterns across the dataset (Figure 4). PC1 primarily differentiated samples based on phenolic richness and antimicrobial potency. Ultrasound-assisted extracts (HSLUAE, HSSUAE) clustered positively along this axis, associated with elevated levels of quercetin, epicatechin, caffeic acid, and rosmarinic acid, alongside enhanced activity against *S. aureus*, *L. monocytogenes*, and *E. coli*. This suggests that UAE facilitates the recovery of bioactive flavonoids and phenolic acids with broad-spectrum antimicrobial effects.

PC2 further separated samples according to genotype-specific traits. The LV 585 extracts (HSLVCE, HSLVUAE) occupied distinct positions in the positive PC2 region, driven by higher contributions from resveratrol, β-resorcylic acid, and activity against *C. parapsilosis* and *S. flexneri*. These associations indicate that genotype LV 585 may possess unique phenolic signatures with antifungal and Gram-negative targeting potential.

The biplot vectors revealed strong correlations between specific phenolic compounds and microbial inhibition. Rosmarinic, resveratrol, ferulic, β-resorcylic, and coumaric acids aligned with *E. coli* and *C. albicans*, positioning HSLUAE and HSLCE near these axes. Quercetin grouped with Gram-positive strains (*S. aureus*, *B. cereus*, *L. monocytogenes*, *C. perfringens*), corresponding to the location of HSLVCE. Gallic and caffeic acids aligned with *P. aeruginosa* and *H. influenzae*, near HSSUAE and HSLVUAE. In contrast, *C. parapsilosis* and *S. flexneri* projected toward the lower quadrant, near HSSCE and away from the principal phenolic vectors.

Overall, PCA clearly distinguished the two extraction methods. Conventionally extracted samples (HSSCE, HSLVCE, HSLCE) clustered near the origin or lower quadrants, reflecting intermediate phenolic content and moderate antimicrobial activity. Ultrasound-assisted extracts (HSSUAE, HSLVUAE, HSLUAE) showed more extreme positions along both components, indicating stronger compositional shifts and enhanced bioactivity. These findings confirm that both genotype and extraction strategy significantly shape the phenolic and antimicrobial profiles of Romanian hemp seed extracts, supporting their potential as natural antimicrobial agents and synergistic enhancers of conventional therapies.

Principal Component Analysis (Figure 5) was employed to investigate the relationships between individual polyphenols identified in *Cannabis sativa* seed extracts and the antimicrobial responses of microbial strains treated with amoxicillin and miconazole. The first two principal components—PC1 (32.55%) and PC2 (25.91%)—explained 58.46% of the total variance, revealing distinct compositional and functional patterns across the six analyzed extracts. PC1 primarily distinguished samples based on phenolic enrichment and synergistic antimicrobial activity. Ultrasound-assisted extracts (HSLUAE, HSSUAE, HSLVUAE) clustered positively along this axis, closely associated with quercetin, epicatechin, caffeic acid, and rosmarinic acid. These compounds correlated with enhanced inhibition of *S. aureus*+A, *L. monocytogenes*+A, and *B. cereus*+A, suggesting that UAE facilitates the recovery of bioactive flavonoids with synergistic effects when combined with conventional antimicrobial agents.

PC2 further separated the samples according to genotype-specific traits and selective microbial targeting. LV 585 extracts (HSLVCE, HSLVUAE) aligned with resorcylic, ferulic, and coumaric acids, showing proximity to *C. albicans*+M and *S. flexneri*+A, indicating potential antifungal and Gram-negative activity. In contrast, HSSCE and HSSUAE clustered near gallic and catechol vectors, associated with *P. aeruginosa*+A and *H. influenzae*, reflecting a distinct phenolic profile and microbial response. The biplot also revealed that rosmarinic, resveratrol, ferulic, β-resorcylic, and coumaric acids contributed most strongly to the variation associated with *E. coli*+A, *C. albicans*+M and partially *B. cereus*+A. Conversely, *S. flexneri*+A projected negatively along PC1, indicating an inverse relationship with these phenolics. *S. typhimurium*+A and *C. perfringens*+A aligned more closely with caffeic and gallic acids, suggesting a distinct response pattern.

Gram-positive strains (*S. aureus*+A, *B. cereus*+A, *L. monocytogenes*+A) clustered along PC2 near quercetin, indicating a specific phenolic association within this axis. These groupings suggest that different polyphenol classes are linked to distinct antimicrobial synergistic profiles.

Across both PCA models (Figure 4 and Figure 5), a clear pattern shows the role of gallic acid in influencing antimicrobial or synergistic activity. Despite its relatively high levels in HSSUAE, the only extract with notable gallic acid, it does not significantly correlate with microbial inhibition or improved antibiotic response. In both biplots, gallic acid loads toward the lower-left, aligning with strains like *P. aeruginosa*, *H. influenzae*, *S. flexneri*, and *C. parapsilopsis*—organisms with weaker or inconsistent responses. This position places gallic acid outside the principal phenolic axes dominated by rosmarinic/resveratrol/ferulic and ferulic acids, as well as quercetin, suggesting that it does not contribute substantially to the biological variance captured by the principal components. From a statistical view, gallic acid’s limited influence is due to its restricted occurrence. It appears in only one extract (HSSUAE) and lacks the shared covariance required to shape the multivariate structure in PCA, which emphasises variables present across many samples. Conversely, phenolics such as rosmarinic, ferulic, resveratrol, and quercetin are present in multiple extracts and strongly co-vary with antimicrobial effects, thus dominating the principal components. PCA treats gallic acid mainly as a sample-specific marker rather than a factor influencing biological activity. Biologically, while gallic acid has shown antimicrobial and efflux pump-modulating properties in some systems [25], its activity tends to be modest, strain-dependent, and often overshadowed by hydroxycinnamic acids and flavonols, which have broader and more potent antimicrobial effects. Gallic acid may also have neutral or antagonistic interactions when mixed with other phytochemicals, especially alongside more active compounds like rosmarinic or ferulic acids [26]. This could explain why HSSUAE, with high gallic acid levels, clusters with strains exhibiting minimal antimicrobial responses in both PCA models. Overall, the combined chemical, biological, and multivariate data suggest gallic acid plays a limited role in the antimicrobial and synergistic effects observed here. Its lack of impact is not due to an absence of activity but because (i) it’s found in only one extract, (ii) it has lower antimicrobial potency compared to main phenolics, and (iii) it does not contribute significantly to the shared variance shaping the principal components.

Therefore, gallic acid remains statistically and functionally secondary in both PCA analyses, with the main antimicrobial effects driven by rosmarinic acid, resveratrol, quercetin, and ferulic acid.

Overall, the PCA biplot highlights the significant influence of the extraction method on the biological behaviour of the samples. Conventionally extracted samples (HSSCE, HSLVCE, HSLCE) tended to cluster near strain–antibiotic vectors associated with moderate synergistic effects. In contrast, ultrasound-assisted extracts (HSSUAE, HSLVUAE, HSLUAE) aligned more closely with axes linked to the strongest synergistic responses—*E. coli*+A, *C. albicans*+M, and *P. aeruginosa*+A. This pattern underscores UAE’s superior capacity to release phenolic acids and flavonoids that enhance antimicrobial activity, supporting its use in producing extracts with higher concentrations of synergistically active compounds.

## 3. Discussion

Plant phenolics are essential dietary components that exhibit significant antioxidant activity and other health benefits [27]. Phenolic acids are known for their antimicrobial properties and their use as food preservatives [28]. Their antimicrobial effectiveness is strongly influenced by their chemical structure—particularly the length of the saturated side chain and the number and positions of substituents on the benzene ring [27]. Hydroxybenzoic and hydroxycinnamic acids exhibit different antimicrobial behaviours depending on the arrangement of their hydroxyl and methoxy groups [29].

Flavonoids are versatile plant compounds that regulate growth, provide pigmentation, and protect against oxidative and UV damage [30]. Their structure—comprising two aromatic rings connected by a three-carbon bridge with phenolic hydroxyl groups—confers strong antioxidant properties and the ability to absorb harmful UV radiation. Due to their bioactive properties, flavonoids are valued in the nutraceutical, pharmaceutical, cosmetic, and medical fields. They protect the human body from oxidative stress [31]. They may lower the risk of various chronic and degenerative diseases, including cancer, diabetes, cardiovascular conditions, neurodegenerative diseases, and inflammatory disorders [32,33].

Stilbenes represent another group of bioactive compounds recognised for their potent antioxidant and anti-inflammatory properties. Additionally, they demonstrate notable antimicrobial effects, inhibiting the growth of bacteria, fungi, and viruses [34].

Our values are consistent with those of Babiker et al., who investigated the effects of roasting times (7, 14, and 21 min) at 160 °C on hemp seed composition, colour attributes, bioactive compounds, and fatty acid profiles. They reported that unroasted hemp seeds contain 4.98 mg/100 g of gallic acid, 0.71 mg/100 g of caffeic acid, 0.40 mg/100 g of quercetin, and 0.05 mg/100 g of resveratrol [35]. Menga and colleagues studied the nutritional and nutraceutical properties of seeds from two monoecious hemp cultivars (Carmaleonte and Codimono) and one dioecious cultivar (CS), finding that whole seeds contained epicatechin acid ranging from 47.2 to 62.1 µg/g, caffeic acid between 2.00 and 2.20 µg/g, and ferulic acid from 0.7 to 2.9 µg/g [36]. Przybylska-Balcerek et al. analysed polyphenols, fatty acids, and terpenes in Henola hemp seeds based on fertilisation method, noting lower concentrations in their control samples compared with our observations: caffeic acid at 0.12–0.17 µg/100 g, coumaric acid at 1.46–2.29 µg/100 g, ferulic acid at 1.62–2.13 µg/100 g, and quercetin at 17.16–18.21 µg/100 g [37].

Corbin et al. demonstrated that optimised and validated UAE extraction conditions for flaxseed yielded higher amounts of secoisolariciresinol diglucoside, herbacetin diglucoside, and hydroxycinnamic acid glucosides than previously reported methods [38].

Overall, the phenolic profile of *Cannabis sativa* seeds was primarily influenced by factors such as the year of production, plant genotype [36,39], and agronomic practices, including fertilisation and harvesting strategies [40].

Research involving clinical bacterial isolates has demonstrated that crude plant extracts may act as sources of resistance-modifying constituents and can improve the efficacy of conventional antibiotics against multidrug-resistant strains [41]. Several mechanisms contribute to antibiotic resistance, including alterations in membrane permeability that reduce antibiotic entry or increase antibiotic removal via efflux pumps. Additionally, bacteria may inactivate the antibiotic, modify its molecular target, or utilise alternative metabolic pathways, including entering a dormant state, secreting target-protecting proteins, and regulating metabolism and initiating self-repair systems, which together constitute the bacterial defence system against antibiotics [42,43,44,45].

The PCA results indicate that the antimicrobial behaviour of the extracts is strongly influenced by the distribution of specific polyphenols rather than by their total phenolic content. The alignment of *E. coli* and *C. albicans* with rosmarinic, resveratrol, ferulic, β-resorcylic and coumaric acids—together with the clustering of HSLUAE and HSLCE—suggests that extracts rich în these compounds exhibit enhanced antimicrobial effects. This interpretation aligns with the extensive evidence of rosmarinic acid and rosemary-derived caffeic acid derivatives’ activity against *E. coli*, *P. aeruginosa*, *Salmonella* spp., and *C. albicans*, including their antibiofilm effects [46]. Likewise, ferulic acid and its derivatives have demonstrated membrane-disrupting and antibiofilm activities in *E. coli* and *P. aeruginosa* [47], while resveratrol shows broad-spectrum efficacy against *Candida* spp. and various Gram-negative and Gram-positive bacteria [48]. Therefore, the placement of these phenolics explains the robust microbial responses observed in HSLUAE and HSLCE.

A distinct second axis emerges around quercetin, which groups with Gram-positive bacteria such as *S. aureus*, *B. cereus*, *L. monocytogenes* and *C. perfringens*. This arrangement reflects the known antimicrobial mechanisms of quercetin, including disruption of peptidoglycan synthesis, damage to the cytoplasmic membrane and inhibition of quorum sensing [49]. The position of HSLVCE within this region suggests that its activity is predominantly driven by flavonol-rich chemistry rather than by the rosmarinic/resveratrol profile.

In contrast, *P. aeruginosa* and *H. influenzae* cluster near gallic and caffeic acids, together with HSSUAE and HSLVUAE, indicating phenolic profiles dominated by simpler hydroxybenzoic and hydroxycinnamic acids. Although these compounds exhibit known antimicrobial and, occasionally, synergistic effects—particularly when combined with ferulic acid [50]—their contribution here appears weaker or more strain-specific, in agreement with the strong intrinsic resistance mechanisms of these Gram-negative species. These plant-derived phytochemicals are known to have antibacterial activity; they can disrupt intracellular protein–protein interactions [51], influence the host’s immune response [52], interfere with bacterial signal and cell division [53], and ultimately promote programmed cell death [54].

Finally, *C. parapsilopsis* and *S. flexneri* show separate responses from the major phenolic vectors, suggesting that their susceptibility to the extracts is lower or less dependent on polyphenolic composition. The marked displacement of HSSUAE along the negative PC2 reinforces the idea of a unique or less-active phenolic profile. Overall, the PCA highlights two principal phenolic-driven axes: (i) a rosmarinic/resveratrol/ferulic axis associated mainly with *E. coli* and *C. albicans*, and (ii) a quercetin axis linked to Gram-positive bacteria. These findings support a model in which individual polyphenols selectively and complementarily influence antimicrobial activity, identifying rosmarinic acid, resveratrol, quercetin and ferulic acid as key contributors to the observed patterns.

The PCA biplot of phenolic composition and synergistic antimicrobial responses shows that the extracts’ chemical profiles strongly influence their ability to potentiate the action of amoxicillin and miconazole. On the positive side of PC1, rosmarinic acid, resveratrol, ferulic acid, β-resorcylic acid, and coumaric acid clustered with *E. coli *+ A and *C. albicans *+ M, indicating that extracts rich in these compounds show enhanced sinergic with amoxicillin in bacteria and with miconazole in *C. albicans*. This relationship corresponds with prior research indicating that rosmarinic acid and related caffeic acid derivatives synergise with β-lactam antibiotics, including amoxicillin, by disrupting membrane integrity, inducing oxidative stress, and interfering with cell wall enzymes [55]. Resveratrol confirms its role as an azole-potentiating compound, supporting reports that it enhances fluconazole and other azoles by inhibiting hyphal development and ergosterol biosynthesis in *C. albicans* [56].

The placement of HSLUAE and HSLCE near this phenolic axis indicates that these extracts possess a rosmarinic–resveratrol–ferulic chemical signature, which is responsible for their strong synergistic behaviour. In contrast, HSLVCE, positioned near quercetin and the Gram-positive strains *S. aureus *+ A, *L. monocytogenes *+ A, *B. cereus *+ A and *S. typhimurium *+ A, reflects a second phenolic interaction pattern dominated by flavonols. This is consistent with evidence showing that quercetin enhances β-lactam activity in Gram-positive bacteria by inhibiting β-lactamases, disrupting peptidoglycan synthesis, and altering membrane integrity [57].

On the negative side of PC1, *P. aeruginosa *+ A and *H. influenzae *+ A clustered with gallic and caffeic acids, suggesting weaker or strain-specific synergistic responses typical of these simpler phenolic acids. Their behaviour corresponds with literature that describes moderate or conditional synergistic effects of gallic and caffeic acids, which are often hindered by the robust inherent resistance mechanisms of Gram-negative bacteria [15].

The separation of *C. parapsilosis *+ M and *S. flexneri *+ A toward the lower-left quadrant suggests that their inhibitory effects rely more on the intrinsic action of miconazole or amoxicillin than on polyphenolic synergism.

Taken together, the PCA supports a dual-axis model of phenolic–antimicrobial synergism: (i) a rosmarinic/resveratrol/ferulic axis enhancing amoxicillin activity against *E. coli* and boosting miconazole action in *C. albicans*, and (ii) a quercetin axis driving synergistic interactions in Gram-positive species. Specific phenolic structures, not total phenolic content, determine extracts’ ability to improve antimicrobial efficacy. Key candidates for future synergy include rosmarinic acid, resveratrol, ferulic acid, and quercetin.

The antimicrobial activity of the hemp seed extracts was closely related to their individual phenolic profiles, as revealed by HPLC and confirmed by PCA. UAE extracts, which contained substantially higher levels of epicatechin, quercetin, rosmarinic acid, resveratrol, ferulic acid, and β-resorcylic acid, consistently showed stronger inhibition of both bacteria and yeasts. These extracts also produced the most pronounced effects in the heatmap, MIC data, and synergy metrics, indicating that antimicrobial potency was determined by the quality and distribution of key phenolics rather than total phenolic content. In particular, strains such as *S. pyogenes*, *S. aureus*, *L. monocytogenes*, *B. cereus*, *E. coli*, and *C. albicans*—which exhibited strong inhibition—clustered near the main phenolic vectors in the PCA, aligning their biological response with the chemical signatures of the most active extracts.

PCA further distinguished two dominant phenolic activity patterns. The first, driven by rosmarinic acid, resveratrol, ferulic acid, and coumaric acid, aligned with *E. coli*, *C. albicans*, and their combination treatments, explaining the high inhibition and substantial % enhancement seen particularly in *C. albicans* + miconazole. The second axis, dominated by quercetin, grouped with Gram-positive bacteria, matching the higher intrinsic and antibiotic-enhancing effects observed for HSLVCE and other quercetin-rich extracts. In contrast, *P. aeruginosa* and *H. influenzae* aligned with gallic and caffeic acids and showed only additive behaviour, consistent with their intrinsic resistance mechanisms. Overall, the combined HPLC, antimicrobial assays, and PCA results demonstrate that specific phenolic structures—especially rosmarinic acid, resveratrol, ferulic acid, and quercetin—drive the antimicrobial and synergistic activity of hemp seed extracts, with UAE extraction producing the most bioactive profiles.

It should be noted that LC–MS-based phenolic profiling does not allow absolute discrimination between positional isomers with identical molecular masses, such as β-resorcylic acid (2,4-dihydroxybenzoic acid) and 2,3-dihydroxybenzoic acid, without compound isolation and spectroscopic confirmation. In the present study, compound assignment relied on retention time matching and UV–MS characteristics of analytical standards, which provides a high level of confidence but does not replace structural elucidation by NMR. In addition, hydroalcoholic extraction may co-extract other polar constituents, including organic acids, sugars, peptides, or minor lipid fractions. While such compounds may contribute to baseline antimicrobial effects, PCA demonstrated that antimicrobial and antibiotic-enhancing activities were specifically associated with phenolic distribution patterns—particularly rosmarinic acid, resveratrol, ferulic acid, and quercetin—rather than with extract complexity or total solubilised material. This indicates that phenolics are the principal contributors to the bioactivity trends observed.

## 4. Materials and Methods

### 4.1. Chemicals

Formic acid, acetonitrile, and ethanol were obtained from Merck KGaA (Darmstadt, Germany). All reagents used for the chemical analysis were of analytical grade. All culture media used in microbiological analyses were purchased from Oxoid Limited, Thermo Fisher Scientific Inc. (Waltham, MA, USA).

### 4.2. Plant Material

All varieties and lines were developed and grown at the Lovrin Agricultural Research and Development Station (45°57′03″ N, 20°46′32″ E). The hemp seed cultivars assessed were Lovrin 110 (HSL) and Silvana (HSS), and the LV 585 (HSLV) line is currently undergoing certification. Before analysis, seeds were milled in a Grindomix GM200 (Retsch GmbH, Haan, Germany), and the resulting meal was used immediately.

### 4.3. Preparation of Plant Extract

Operating conditions for ultrasound-assisted extraction (UAE) and conventional extraction (CE) were determined based on previous reports on these techniques [58,59] and from our own [60] trials with various matrices. For each cultivar, milled seeds were combined with a 70% (*v*/*v*) ethanol solution to form the extraction suspension. The mixtures were then processed using two protocols: a standard conventional extraction (CE) and an alternative ultrasound-assisted extraction (UAE) method.

Conventional solvent extraction (CE) and ultrasound-assisted extraction (UAE)

The preparation of the extracts, both by the conventional method and by ultrasound-assisted extraction, was carried out according to the protocol described by Floares et al. [61]. After the extraction step, the mixture was filtered through filter paper, and the resulting filtrate was stored at 4 °C until analysis. The extraction was performed in three repetitions.

### 4.4. Individual Polyphenols by HPLC Analysis

Compound identification was performed using retention time matching, UV spectra, and MS data compared with authentic standards; therefore, assignments should be regarded as putative within the limits of LC–MS-based phenolic profiling. HPLC quantified individual polyphenols–MS using a Shimadzu 2010 EV system (Shimadzu, Kyoto, Japan) fitted with an SPD-10A UV detector and an MS detector, and equipped with an EC 150/2 NUCLEODUR C18 Gravity SB column (150 × 2 mm, 5 μm; Macherey-Nagel GmbH & Co. KG, Dueren, Germany). The mobile phases were: A—water acidified with formic acid to pH 3; B—acetonitrile similarly acidified to pH 3. The gradient was programmed as follows: 0.01–20 min, 5% B; 20.01–50 min, 5–40% B; 50–55 min, 40–95% B; 55–60 min, 95% B. The eluent flow rate was 0.2 mL/min, and the column temperature was maintained at 20° C. UV monitoring was carried out at 280 and 320 nm. Calibration curves were prepared over the 20–50 μg/mL range [62]. Method validation parameters were evaluated to ensure the reliability of the HPLC analysis. Linearity was confirmed using external calibration curves for each phenolic standard, expressed by the following equations: gallic acid, f(x) = 6.04661 × 10^−10^x − 3.04827; caffeic acid, f(x) = 7.36054 × 10^−10^x + 3.55894; epicatechin, f(x) = 3.43873 × 10^−10^x + 4.99711; β-resorcylic acid, f(x) = 1.45563 × 10^−10^x + 3.75512; p-coumaric acid, f(x) = 4.34018 × 10^−10^x + 3.48454; ferulic acid, f(x) = 8.1887 × 10^−10^x + 3.79724; rosmarinic acid, f(x) = 2.21544 × 10^−10^x + 1.3627; resveratrol, f(x) = 4.37174 × 10^−10^x + 1.3537; and quercetin, f(x) = 1.3719 × 10^−10^x + 3.97487. Precision showed RSD values below 5%, accuracy ranged between 92% and 105% recovery, selectivity was ensured by retention time and UV–MS matching with standards, robustness was confirmed across independent extractions, and LOD/LOQ were estimated using signal-to-noise ratios of 3 and 10, respectively.

All reagents and solvents were of analytical grade, and each sample was analysed in triplicate.

### 4.5. Antimicrobial Activity

The antimicrobial activity of CS used in this study was evaluated through broth microdilution against various ATCC strains, categorised into Gram-positive and Gram-negative groups. The Gram-positive ATCC strains tested were *Streptococcus pyogenes* (ATCC 19615), *Staphylococcus aureus* (ATCC 25923), *C. perfringens* (ATCC 13124), *Listeria monocytogenes* (ATCC 19114), and *Bacillus cereus* (ATCC 10876). And the Gram-negative strains taken into the study were: *Pseudomonas aeruginosa* (ATCC 27853), *Shigella flexneri* (ATCC 12022), *Escherichia coli* (ATCC 25922), *Salmonella typhimurium* (ATCC 14028), *Haemophilus influenzae type B* (ATCC 10211), and two fungal strains *Candida albicans* (ATCC 10231) and *Candida parapsilopsis* (ATCC 22019).

A 10^−3^ dilution (0.5 McFarland standard) of the fresh culture in Brain Heart Infusion (BHI) broth (Oxoid, CM1135) was used to test the antimicrobial activity of the hemp seed extracts, as our previous study describes [63]. The extracts were added directly to the bacterial suspension at different quantities (0.009 mg/mL; 0.0135 mg/mL; 0.018 mg/mL; 0.0225 mg/mL; 0.045 mg/mL; 0.0675 mg/mL). The concentrations tested were chosen based on previous research and a literature review to cover a broad spectrum of concentrations and identify possible MIC values.

The MIC, defined as the lowest concentration of the compound that yields no visible microbial growth, was determined by measuring optical density (OD) using the spectrophotometric method. Two indicators, BGP (bacterial growth percentage) and BIP (bacterial inhibition percentage), were calculated for interpreting the results, based on the following Formulas (1) and (2):

(1) $$BGP\;(\%)=\frac{OD\;sample}{OD\;negative\;control\;sample}\times 100$$

(2) BIP (%)=100−BGP (%)

where OD sample represents the optical density at 540 nm as a mean value of triplicate readings; OD negative control represents the optical density at 540 nm as a mean value of triplicate readings for the selected bacteria in BHI.

### 4.6. Evaluation of the Capacity to Potentiate the Antibacterial Activity of Amoxicillin by Fold Reduction Index in MIC and % Enhancement

The interaction between *Cannabis sativa* seed extracts and amoxicillin was tested using the checkerboard method, as previously described in the literature [64]. Briefly, into each 96-well plate, 100 µL of a 1.5 × 10^8^ CFU/mL (0.5 McFarland standard) bacterial suspension was inoculated. The antibacterial action of HSLCE, HSLUAE, HSSCE, HSSUAE, HSLVCE, HSLVUAE and amoxicillin/miconazole (Sigma-Aldrich, Merck KGaA, Darmstadt, Germany) mixtures were determined using the Minimum Inhibitory Concentration (MIC) microdilution assay and verified according to CLSI recommendations (Clinical and Laboratory Standard Institute, 2002). The extracts were added directly to the bacterial suspension at different quantities (0.009 mg/mL; 0.0135 mg/mL; 0.018 mg/mL; 0.0225 mg/mL; 0.045 mg/mL; 0.0675 mg/mL). The Clinical and Laboratory Standard Institute (CLSI) recommendations were followed [65].

In all combination experiments, amoxicillin/miconazole was intentionally used at doses below the minimum inhibitory concentration established for each ATCC strain. These sub-MIC amounts were mixed with 50 µL of DMSO carrying the different extract concentrations to ensure that any observed inhibitory enhancement resulted from extract–antibiotic interactions rather than from the antibiotic alone. Then, the plates were incubated for 24 h at 37 °C in aerobic conditions. All tests were performed in triplicate.

After the incubation period, the OD of each well plate was measured using the spectrophotometric method.

The antimicrobial activity of amoxicillin and miconazole alone was evaluated for each tested strain and used as a reference control in all combination experiments. In order to assess extract-mediated potentiation, antibiotics and antifungals were deliberately applied at sub-inhibitory concentrations that did not produce complete growth inhibition when used alone. Optical density values obtained for antibiotic- or antifungal-only treatments (OD_antibiotic) served as the baseline for calculating percent enhancement and for comparing combination effects relative to the drug alone. A synergy analysis was performed using two complementary metrics: fold reduction in MIC and percent enhancement in inhibition. Fold-reduction was calculated as the ratio of the MIC of the extract alone to the MIC of the extract in combination with a sub-inhibitory concentration of an antibiotic or antifungal. Interpretation thresholds were defined as follows: ≥4-fold = strong synergy, 2–4-fold = moderate synergy, 1-fold = additive interaction, and <1-fold = antagonism.

To evaluate the enhancement in inhibition from the combination treatments, the per cent enhancement metric was calculated using the Formula (3):

% Enhancement = ((OD_antibiotic − OD_combination)/OD_antibiotic) × 100.(3)

Values ≥10% indicated strong enhancement, 5–10% moderate enhancement, and <5% weak or no enhancing effect as presented in Table 4.

### 4.7. Statistical Analysis

The results were expressed as mean ± standard deviation. One-way ANOVA with Tukey’s post hoc test (*p* < 0.05) was carried out using JASP 0.19.3.0. PCA with varimax rotation was performed in OriginPro 2026 to reduce data dimensionality and to identify the main patterns of variation among samples.

## 5. Conclusions

This study demonstrates that the antimicrobial and antibiotic-enhancing activities of hemp seed extracts are determined by their specific phenolic composition rather than by total phenolic content. UAE extraction yielded higher concentrations of key bioactive molecules—particularly rosmarinic acid, resveratrol, ferulic acid, epicatechin, and quercetin—corresponding to stronger inhibition of bacteria and yeasts. MIC reduction, % enhancement, and PCA consistently showed that these compounds drive two major activity patterns: a rosmarinic/resveratrol/ferulic axis linked to strong effects in *E. coli* and *C. albicans*, and a quercetin axis associated with Gram-positive bacteria. The most significant synergistic responses occurred in *S. pyogenes*, *S. aureus*, *B. cereus*, and *C. albicans*, while most Gram-negative strains exhibited additive behaviour. These findings identify hemp seeds—particularly when extracted by UAE—as promising natural antimicrobial agents and effective adjuvants for conventional antibiotics and antifungals.

## Figures and Tables

**Table 1:** The individual profile of polyphenols detected using the HPLC method.

Individual Polyphenol Compounds	Class	Ret. Time (min.)	*m*/*z*	HSLCE	HSSCE	HSLVCE	HSLUAE	HSSUAE	HSLVUAE
				Concentration µg/g					
Gallic	Phenolic acids	2.93	169	nd *	nd	nd	nd	178.1 ± 0.41	nd
Epicatechin	Flavonoids	6.67	289	nd	18.45 ± 0.22 ^a^	nd	42.00 ± 0.33 ^b^	55.36 ± 0.27 ^c^	72.04 ± 0.44 ^d^
Caffeic	Phenolic acids	7.95	179	nd	nd	4.79 ± 0.08 ^b^	nd	3.60 ± 0.09 ^a^	nd
β-resorcylic	Phenolic acids	9.93	153	nd	10.27 ± 0.30 ^a^	nd	18.02 ± 0.62 ^b^	nd	nd
Coumaric	Phenolic acids	12.62	163	3.50 ± 0.25 ^a^	4.32 ± 0.16 ^b^	3.50 ± 0.22 ^a^	6.80 ± 0.50 ^c^	nd	nd
Ferulic	Phenolic acids	14.91	193	3.85 ± 0.15 ^a^	3.92 ± 0.11 ^ab^	3.81 ± 0.24 ^a^	4.72 ± 0.12 ^b^	nd	3.81 ± 0.58 ^a^
Rosmarinic	Phenolic acids	23.56	359	7.02 ± 0.23 ^c^	1.82 ± 0.24 ^a^	3.57 ± 0.23 ^b^	11.24 ± 0.15 ^d^	1.45 ± 0.57 ^a^	3.50 ± 0.32 ^b^
Resveratrol	Stilbenes	27.18	227	6.11 ± 0.20 ^c^	1.41 ± 0.19 ^a^	5.91 ± 0.18 ^c^	13.29 ± 0.38 ^e^	4.68 ± 0.46 ^b^	7.15 ± 0.54 ^d^
Quercitine	Flavonoids	30.90	301	5.79 ± 0.24 ^b^	4.25 ± 0.17 ^a^	4.15 ± 0.15 ^a^	9.99 ± 0.63 ^c^	5.66 ± 0.36 ^b^	20.53 ± 0.65 ^d^

hemp seed conventional extraction (CE): Lovrin 110—HSLCE, Silvana—HSSCE, Lovrin 585—HSLVCE; hemp seed ultrasound-assisted extraction (UAE): Lovrin 110—HSLUAE, Silvana—HSSUAE, Lovrin 585—HSLVUAE. *—not detected. All results are reported as the mean of three replicates ± standard deviation (SD) (*n* = 3). Based on ANOVA analysis, differing lower-case letters (a–e) within the same row indicate statistically significant differences between samples at *p* < 0.05.

**Table 2:** Fold reduction in MIC values for hemp seed extracts combined with amoxicillin or miconazole compared to the single extract.

Fold Reduction	*S. pyogenes*	*S. aureus*	*L. monocytogenes*	*B. cereus*	*C. perfringens*	*S. flexneri*	*E. coli*	*P. aeruginosa*	*S. typhimurium*	*H. influenzae*	*C. albicans*	*C. parapsilopsis*
HSLCE+A	2.0	1.0	1.0	1.3	1.0	1.0	1.0	1.0	1.0	1.5	1.0	1.5
HSLUAE+A	0.7	1.0	1.0	1.7	1.0	1.0	1.0	1.0	1.0	1.5	1.0	1.0
HSSCE+A	1.3	0.7	1.0	0.5	1.0	1.0	1.0	1.0	1.0	1.0	1.0	1.0
HSSUAE+A	3.8	2.0	1.0	1.5	1.0	1.0	1.0	1.0	1.0	1.0	1.0	1.0
HSLVCE+A	3.3	3.3	1.0	1.3	1.0	1.0	1.0	2.0	1.0	1.0	1.0	1.0
HS-VUAE+A	1.7	2.5	1.0	1.0	1.0	1.0	1.0	2.0	1.0	1.0	1.0	1.0

**Table 3:** Percent enhancement of antimicrobial and antifungal activity produced by combining hemp seed extracts with amoxicillin (bacteria) or miconazole (yeasts).

Enhancement%	*S. pyogenes*	*S. aureus*	*L. monocytogenes*	*B. cereus*	*C. perfringens*	*S. flexneri*	*E. coli*	*P. aeruginosa*	*S. typhimurium*	*H. influenzae*	*C. albicans*	*C. parapsilopsis*
HSLCE+A	4.6	0.3	10.5	9.0	90.0	63.5	79.0	29.3	48.6	16.8	70.6	13.9
HSLUAE+A	0.9	−2.4	11.6	2.3	12.1	75.5	75.2	29.2	51.6	22.6	74.5	31.2
HSSCE+A	3.5	30.4	51.3	43.2	60.6	58.3	71.8	47.9	55.3	70.2	73.6	43.6
HSSUAE+A	0.0	14.0	5.9	4.4	84.0	65.4	62.2	20.6	63.1	38.0	57.8	40.7
HSLVCE+A	0.4	1.8	50.8	6.4	30.0	55.5	57.9	10.8	60.4	35.5	67.9	6.3
HSLVUAE+A	5.4	27.1	57.3	−3.3	50.6	44.6	52.9	8.3	42.4	30.1	51.7	35.1

**Table 4:** Formulas used and interpretation rules for Fold reduction and % Enhancement.

	Formula	Interpretation Rule
Fold reduction	MIC extract ÷ MIC (extract + antibiotic/antifungal)	≥4 = strong synergy; 2–4 = moderate synergy; 1 = additive; <1 = antagonism
% Enhancement	((OD antibiotic − OD combination)/OD antibiotic) × 100	≥10% = strong; 5–10% = moderate; <5% = weak/no effect

## Data Availability

The analytical datasets supporting this study are archived within the Interdisciplinary Research Platform of the University of Life Sciences “King Mihai I” of Timișoara (USVT).
